# Integrative analysis and validation of dysregulated long non‐coding RNAs in colon cancer

**DOI:** 10.1111/jcmm.14974

**Published:** 2020-01-20

**Authors:** Qun Zhang, Yinzhu Bian, Yiping Zhu, Li Wan, Linghui Kong, Jing Hu, Mi Yang, Li Li, Baorui Liu, Xiaoping Qian

**Affiliations:** ^1^ The Comprehensive Cancer Center of Nanjing Drum Tower Hospital Medical School of Nanjing University Clinical Cancer Institute of Nanjing University Nanjing China; ^2^ The Comprehensive Cancer Center of Nanjing Drum Tower Hospital Clinical College of Nanjing Medical University Nanjing China; ^3^ The Comprehensive Cancer Center of Nanjing Drum Tower Hospital Clinical College of Traditional Chinese and Western Medicine Nanjing University of Chinese Medicine Nanjing China

**Keywords:** CASC21, colon cancer, lncRNAs, metastasis, proliferation

## Abstract

It is an increasing evidence that long non‐coding RNAs (lncRNAs) are involved in tumour initiation and progression. Here, we analysed RNA‐sequencing data from the Cancer Genome Atlas (TCGA) datasets. Totally, 1176lncRNAs, 245miRNAs and 2081mRNAs were identified to be differentially expressed (DE) in colon cancer tissues compared with normal tissues. CASC21, a novel lncRNA located in 8q24.21 locus, was significantly overexpressed in 30 colon cancer tissues compared with matched normal tissues by qRT‐PCR assay. CASC21 tended to higher expression as the increase of the tumour‐node‐metastasis (TNM) classification. Functionally, CASC21 promoted cell proliferation by regulating cell cycle and enhanced tumour metastasis by epithelial‐mesenchymal transition (EMT) in colon cancer. Mechanism study indicated that CASC21 might be involved in activating WNT/β‐catenin pathway in colon cancer. In addition, we also built a competing endogenous RNA (ceRNNA) network by bioinformatic analysis using TCGA datasets. Together, our results not only provide novel lncRNAs as potential candidates for further study but also prove that CASC21 is an oncogenic regulator through activating WNT/β‐catenin signalling in colon cancer.

## 1 INTRODUCTION

Colon cancer is the third most common malignancy worldwide.^1^ The incidence of colon cancer is still increasing in most developing countries due to lack of early screening strategies.^2^ Recently, combined therapies including surgery, chemotherapy, targeted therapy and radiotherapy have prolonged overall survival (OS) of patients with colon cancer. However, distant metastasis and drug resistance are still major causes for poor therapeutic efficacy in patients with colon cancer. Recently, some dysregulated lncRNAs were identified to be diagnostic biomarkers or therapeutic targets. A growing body of evidence has indicated that lncRNAs play crucial roles in tumour growth and metastasis including colon cancer.^3^, ^4^, ^5^, ^6^ Thus, increasing dysregulated lncRNAs have been demonstrated to be associated with prognosis of cancer patients and even predict clinical responses to cancer therapy. In our previous reports, we proved that lncRNA KCNQ1OT1 is up‐regulated in and serves as an oncogene while PGM5‐AS1 is down‐regulated and serves as a tumour suppressor in colorectal cancer.

It is reported that lncRNAs exert its function by diverse mechanisms depending on its cellular location.^7^, ^8^, ^9^, ^10^ The 8q24.21 locus harbours several cancer‐related lncRNAs and few protein‐coding genes.^11^ The MYC oncogene, located in this region, contributes to the tumorigenesis in many human cancers including colon cancer.^12^, ^13^ Previous reports demonstrated that lncRNAs mapped to 8q24.21 locus paly potential roles by directly or indirectly interacting with MYC. For instance, CCAT1, CCAT2 and CASC11 are identified to be closely associated with MYC,^14^, ^15^ emphasizing the clinical significance of dysregulated lncRNAs in this region.

In this study, we first identified DElncRNAs and constructed a reliable ceRNA network for further research in colon cancer based on TCGA datasets using bioinformatic analysis. CASC21 is located on chromosome 8q24.21, and it is significantly up‐regulated in colon cancer. However, the biological function of CASC21 in colon cancer remains unknown.

## 2 MATERIALS AND METHODS

### 2.1 TCGA analysis

1. Screening of DE lncRNAs: We downloaded RNA‐sequencing data from TCGA colon cancer datasets including 480 colon cancer and 41 normal tissues. The following exclusion criteria were applied for the selection process: (a) Histological diagnosis was not colon cancer, and (b) data were incomplete. Clinical survival data were also obtained from TCGA. OS was the time from tumour resection to death, loss‐to‐follow‐up or study conclusion. R software and packages were used to analyse the data, and a gene with an absolutely log fold change (FC) ≥2 and false discovery rate (FDR) <0.01 was considered to be differentially expressed. We used the Kaplan‐Meier method to analyse OS, and the log‐rank test was used to analyse differences in OS *P* < .05 was regarded as statistical significance (*P < .05; **P < .01; ***P < .001).
2. Gene set enrichment analysis (GSEA): RNA‐sequencing data from TCGA datasets were analysed, and we divided CASC21 into two groups based on its expression. Then, GSEA was carried out by the GSEA software. The enrichment scores were compared with the enrichment results of 1000 random sequences of the gene set to assess statistical significance.
3. Kyoto Encyclopedia of Genes and Genomes (KEGG) pathway enrichment analysis was performed to analyse functions of DEmRNAs. Construction of ceRNA network: Firstly, DElncRNAs in colon cancer were identified as described above. And DElncRNAs which were not annotated by GENCODE were discarded. Secondly, DElncRNA‐DEmiRNA interactions were predicted by miRcode and starBase v2.0. Thirdly, DEmRNAs targeted by DEmiRNAs were identified by three databases (miRTarBase, miRDB and TargetSscan). Finally, the ceRNA network was visualized with Cytoscape 3.5.1.

### 2.2 Clinical samples and Cell lines

Five colon cancer cell lines (HT29, HCT116, SW480, SW620 and LOVO) were cultured according to the instructions recommended by American Type Culture Collection (ATCC). The colon epithelial cell line NCM460 was purchased from the Chinese Academy of Sciences (China) and cultured according to the manufacture's protocol. 30 paired of clinical tissues (colon cancer and corresponding normal tissues) and 20 colon cancer tissues from 50 patients were obtained from Nanjing Drum Hospital (Nanjing, China). All patients signed informed consent forms.

### 2.3 qRT‐PCR analysis

TRIzol (Invitrogen) reagent was used to extract total RNA. Total RNA was reversely transcribed into cDNA using Thermo Scientific™ EP0733 1st Strand Synthesis Kit. The primer sequences: CASC21, forward: 5′CAGCCTCAGAGGTGCTTATTTAG3′, reverse: 5′AGCAGAGAAATAAGGACCTTGACT3′. GAPDH, forward: 5′TGGGTGTGAACCATGAGAAGT3′, reverse: 5′ TGAGTCCTTCCACGATACCAA‐3′.

### 2.4 RNA sequencing

RNA‐sequencing assay was carried out to detect the expression profiles of lncRNAs and mRNAs using 20 colon cancer tissues at Sangon biotech. RNA labelling and hybridization array assays were performed according to the manufacturer's protocol. Libraries were controlled for quality and quantified using Hiseq 2500 system (Illumina).

### 2.5 Cell transfection

The CASC21 small interfering(siRNA) and negative control siRNAs from Ribo were transfected into HCT116 and SW480 cells using Lipofectamine 3000 (Invitrogen) following the manufacturer's instructions. Cells were collected after 24 hours for assays including Western blot and qRT‐PCR. The CASC21 siRNA sequences: siRNA1#, 5′‐GGTTGTTGCTTCCTAGTCT ‐3′; siRNA3#, 5′‐GCTGAGTTCTACTAGCAAA ‐3′.

For CASC21 stable knockdown, sh‐CASC21 construct was generated by Shanghai GenePharma Co, and then, we transfected HCT116 and SW480 cells with sh‐CASC21. Puromycin with a concentration of 1 μg/mL was used to select cells with CASC21 stable knockdown. After 7 days of filtration with puromycin, qRT‐PCR was used to validate cells with CASC21 stable knockdown, and then, cells were cultured in RPMI 1640 (Hyclone) with 10% foetal bovine serum (FBS) and 0.5 μg/mL puromycin. The CASC21 shRNA sequences are as follows: 5′‐GGTTGTTGCTTCCTAGTCT ‐3′.

### 2.6 Cell proliferation assay

In CCK‐8 assay, 3 × 10^3^ cells were cultured in a 96‐well plate for 24, 48, 72 and 96 hours. Then, each well added 10 μL CCK‐8 solution and incubated for 2 hours. The absorbance was measured at 450 nm for each well.

In colony formation assay, cells were counted and 1 × 10^3^ cells were cultured each well in a 6‐well plate for 14 days. Then, colonies were fixed with 4% paraformaldehyde and stained with 0.1% crystal violet. Finally, the mean colony numbers were calculated.

### 2.7 Transwell assay

Transwell assay (Corning) was used to detect the capacity of colon cancer cell invasion and migration. For cell invasion assay, cells (5 × 10^5^) in 200 μL medium with 1% FBS were added into the upper chamber with Matrigel (Sigma‐Aldrich). Six hundred micro litre medium containing 10% FBS was placed into the lower chamber. Methanol was used to fix cells, and 0.1% crystal violet was used to stain cells. For cell migration assay, the procedure was similar to the invasion assay except for the upper chamber without Matrigel.

### 2.8 Flow cytometric assay

For cell cycle assay, 1 × 10^6^ cells were washed by normal saline (NS) and stained with propidium iodide and then incubated in the dark for 30 minutes. For cell apoptosis assay, 1 × 10^6^ cells were stained with annexin V, fluorescein isothiocyanate (FITC) and incubated in the dark for 5 minutes. Samples were finally analysed by flow cytometry.

### 2.9 Western blotting assay

RIPA extraction reagent (Beyotime) was used to lyse cells. Extracted proteins were then separated using 10% sodium dodecyl sulfate‐polyacrylamide gel electrophoresis (SDS‐PAGE). And proteins were transferred to polyvinylidene fluoride (PVDF) membranes (Millipore). Membranes were then incubated with primary antibodies and corresponding secondary antibodies. The primary antibodies against E‐cadherin (CST), N‐cadherin (CST), β‐actin (CST), CDK4 (CST), CDK6 (CST), CyclinD1 (CST), Bax (CST), Bcl‐2 (CST), β‐catenin (CST), GAPDH (CST), Cleaved caspase‐3 (CST), GSK3β(CST), TCF‐4 (Abcam), c‐myc (Abcam) and MMP7 (Abcam) were used in this study. Signals were visualized by ECL reagent.

### 2.10 Immunofluorescence (IF) and Fluorescence in situ hybridization (FISH)

Cells were harvested and washed by NS and then fixed with 4% formaldehyde for 15 minutes. 0.3% Triton X‐100 was used to permeate cells. Cells were incubated with primer antibodies and then corresponding secondary antibodies. The primer antibodies were E‐cadherin (CST) and N‐cadherin (CST). Finally, DAPI was used to stain cellular nuclei.

For fluorescence in situ hybridization, HCT116 and SW480 cells were fixed and washed. Cells were permeated by 0.3% Triton X‐100. Subsequently, cells were incubated with 20 uM FISH Probe in hybridization buffer at 37°C overnight. Cells were washed by 4 × saline sodium citrate (SSC), 2 × SSC and 1 × SSC. Then, cellular nuclei were stained with DAPI. Finally, fluorescence was visualized with a microscope.

### 2.11 Immunohistochemistry (IHC) assay

Tissue sections were incubated with the primary antibody targeting ki67 (Abcam). Then, they were incubated with corresponding secondary antibody.

### 2.12 Animal experiments

We performed animal experiments according to the experimental animal use guidelines of the National Institutes of Health. Male 5‐ to 6‐week‐old BABL/c nude mice were purchased from the Experimental Animal Center of Nanjing Drum Tower Hospital. For the tumorigenicity assay, 1 × 10^6^ cells were stably transfected with Sh‐CASC21 or negative controls, and they were inoculated subcutaneously into the left groin region of mice. The tumour growth was measured every 3 days. All mice were killed after 15 days, and tumour tissues were used to perform haematoxylin and eosin staining, IHC and TUNEL assays. Tumour volumes = 0.5 × *D* (the longest diameter of the tumour) × *d* ^2^ (the longest diameter of the tumour). For the in vivo metastasis assay, 3 × 10^6^ HCT116 cells stably expressing shCASC21 or negative controls were injected into nude mouse tail vein. These mice were killed after 3 weeks, and the number of metastatic foci was counted.

### 2.13 Statistical analysis

We used R software and packages to analyse the RNA‐sequencing data. SPSS 17.0 was used to data analysis. All data were presented as mean ± SD. Image‐Pro Plus (IPP) software was used to perform quantitative analysis of photographs obtained from immunochemistry, IF and WB. The Kaplan‐Meier method and log‐rank tests were used to analyse the survival curves. Relationships between CASC21 expression and clinicopathologic characteristics were analysed by the chi‐square test. Relationships between the expression levels of CASC21 and CCND1or MYC were analysed by Pearson's correlation coefficient, respectively. The differences between two groups were analysed by a Student t test, and one‐way analysis of variance (ANOVA) was used to compare differences among three or more groups. *P* < .05 was regarded as statistically significant.

## 3 RESULTS

### 3.1 Identification of dysregulated lncRNAs using RNA‐sequencing data from TCGA colon cancer datasets

**Figure 1:**
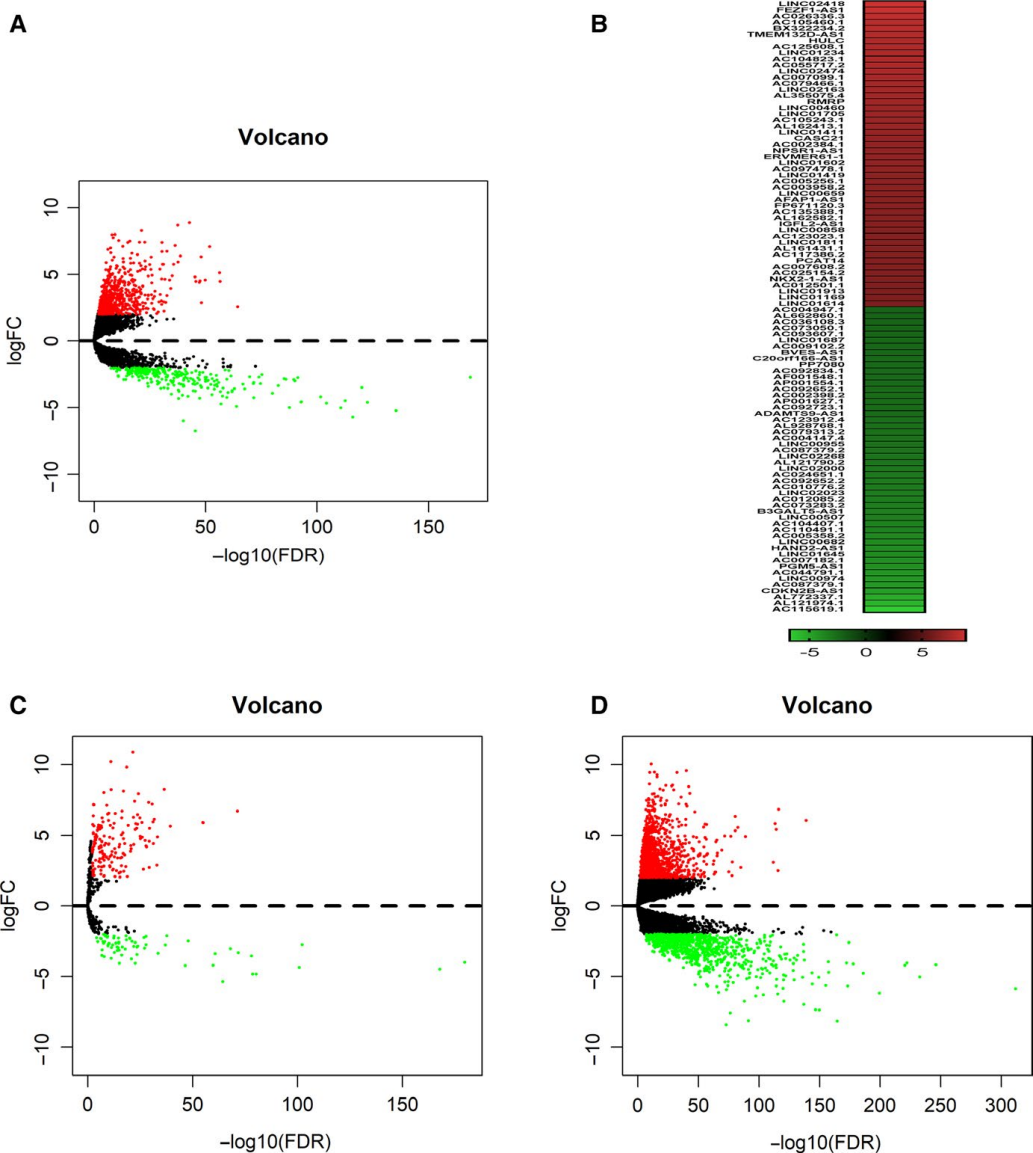
Transcriptome landscape of colon cancer. A, Volcano plot of DElncRNAs identified from TCGA colon cancer datasets. (log fold change ≥2 and *P* < .01); B, The 50 most up‐regulated and down‐regulated DElncRNAs in colon cancer. C, Volcano plot of DEmiRNAs identified from TCGA colon cancer datasets (colon cancer tissues vs normal tissues, log fold change ≥2 and *P* < .01); D, Volcano plot of DEmRNAs identified from TCGA colon cancer datasets (colon cancer tissues vs normal tissues, log fold change ≥2 and *P* < .01)

We identified 1176 DElncRNAs in colon cancer tissues compared with normal tissues using TCGA datasets (Figure 1A, Table S1). The 100 most dysregulated lncRNAs including 50 most up‐regulated and down‐regulated lncRNAs were listed in Figure 1B. Besides, we also identified 245 DEmiRNAs and 2081 DEmRNAs (Figure 1C,D). Using the method mentioned above, we constructed a ceRNA network including 136 lncRNAs, 29 miRNAs and 53 mRNAs (Figure S1). Among them, 10 DElncRNAs, one miRNA and 4 mRNAs were significantly associated with OS in patients with colon cancer (*P* < .05) (Figures S2 and S3). Survival analysis showed that higher expression of AC131571.1, ANO1‐AS2, ITCH‐IT1, ARHGEF26‐AS1, AP004609.1, LINC00491, KCNQ1OT1, MACROD2‐IT1, TSSC1‐IT1 or LINC00355 was correlated to poor OS in patients with colon cancer. Meanwhile, we analysed the potential functions of DEmRNAs involved in the network using DAVID database and KOBAS3.0. KEGG pathway analysis indicated most of the pathways were cancer‐related, implying the most of dysregulated genes we identified in this study might be candidates for cancer research (Table S2).

### 3.2 CASC21 is significantly up‐regulated in colon cancer

**Figure 2:**
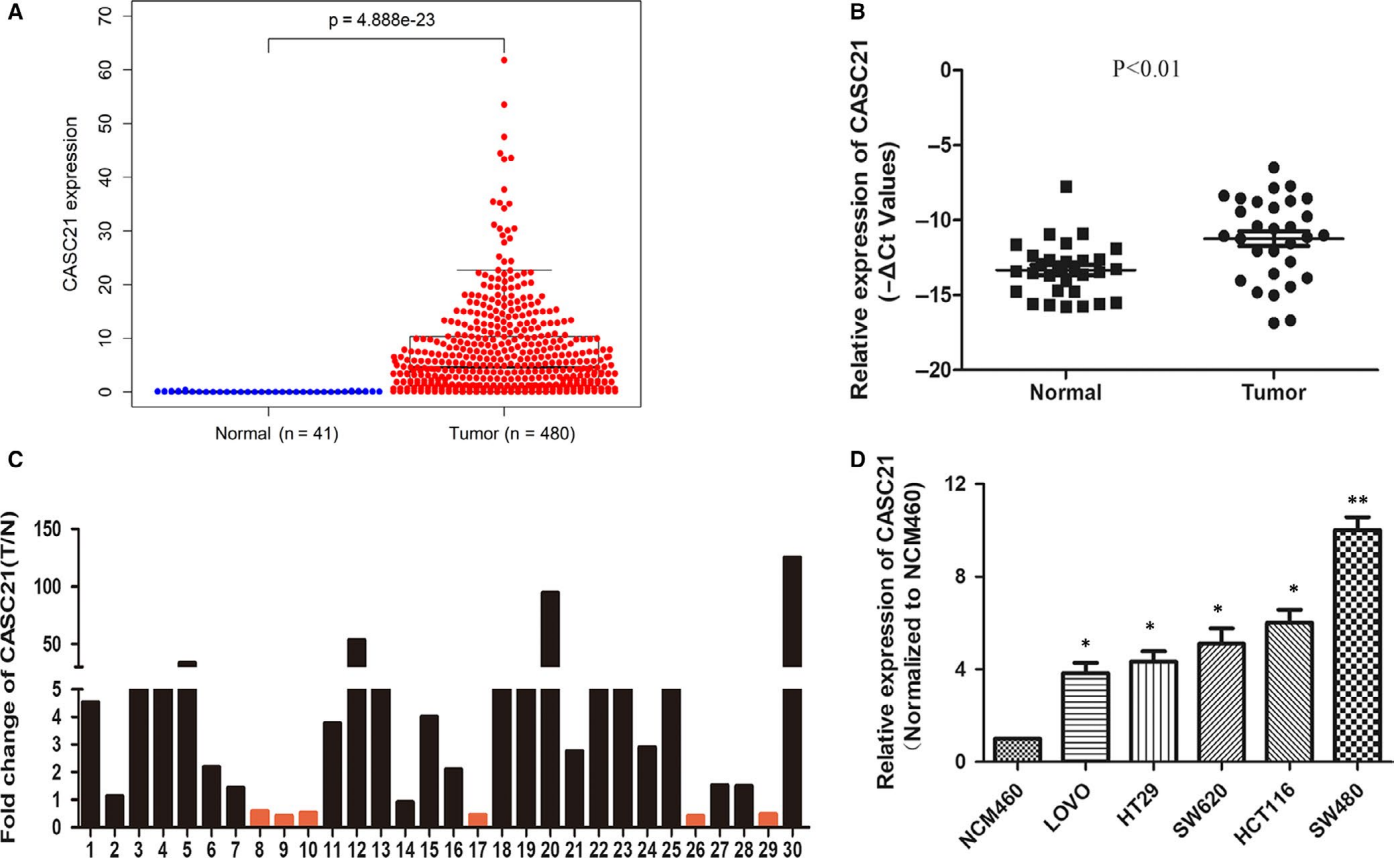
CASC21 is significantly up‐regulated in colon cancer. A, Relative expression of CASC21 in colon cancer tissues compared with normal tissues in TCGA datasets; B, The relative expression of CASC21 was detected by qRT‐PCR using 30 paired of colon cancer and corresponding normal tissues, and GAPDH was used as internal control. Data were presented as ‐ΔCt values. (2^−ΔΔCt^ was used to calculate the relative expression of CASC21. ΔCt = Ct CASC21‐Ct GAPDH, ΔΔCt = ΔCt(cancer tissues)–ΔCt(normal tissues)). C, CASC21 expression was analysed in 30 paired of colon cancer tissues and corresponding normal tissue, and data were presented as the fold change in tumour tissues compared with the matched normal tissues. D, Relative CASC21 expression was analysed in five colon cancer cell lines and one normal colon epithelial cell line, and normalized to GAPDH expression in colon epithelial cell line. (*P < .05, **P < .01)

Bioinformatic analysis of TCGA datasets showed that CASC21 expression was significantly up‐regulated in colon cancer (Figure 2A, *P* = 4.888e‐23). Similarly, our results showed that CASC21 expression was markedly overexpressed in 30 colon cancer tissues compared with matched normal tissues (Figure 2B, *P* < .01). 24/30 (80%) tumour tissues showed an increase in CASC21 expressions in our cohort (Figure 2C). To uncover clinical implications of CASC21, we analysed the relationships between clinical pathological parameters and CASC21 expression. We found a significantly elevated expression of CASC21 in patients with advanced TNM stages (*P* = .025, Table 1). However, no significant differences between CASC21 expression and other clinical pathological parameters were observed in this study due to the limited sample size. Interestingly, we observed CASC21 expression was slightly higher in tumours with mismatch‐proficient (pMMR) than tumours with mismatch‐deficient (dMMR), although the difference did not achieve statistical significance (*P* = .068, Table 1). Meanwhile, in comparison with NCM460 cell line, CASC21 expression was obviously increased in five human colon cancer cell lines (Figure 2D). Of them, HCT116 and SW480 cell lines, with relative higher expression of CASC21, were selected for further functional assays.

**Table 1:** Clinicopathologic characteristics of CASC21 expression in colon cancer patients

Characteristics	CASC21		*P* value
	Low	High	
Ages (y)			
<60	3	5	.409
≥60	12	10	
Gender			
Male	8	6	.464
Female	7	9	
Tumour size (cm)			
≤4	6	6	1.0
>4	9	9	
Differentiation			
Well and moderately	1	1	1.0
Poorly	14	14	
Lymph metastasis			
Yes	4	7	.253
No	11	8	
Tumour stage			
I + II	12	6	**.025** *
III + IV	3	9	
MMR			
dMMR	3	0	**.068**
pMMR	12	15	

Note

*P*‐value <.05 was considered statistically significant.

* Represents *P* < .05.

*P*‐value <.05 or a borderline *P*‐value was marked in bold.

### 3.3 CASC21 knockdown suppresses cell proliferation and induces cell apoptosis in colon cancer

**Figure 3:**
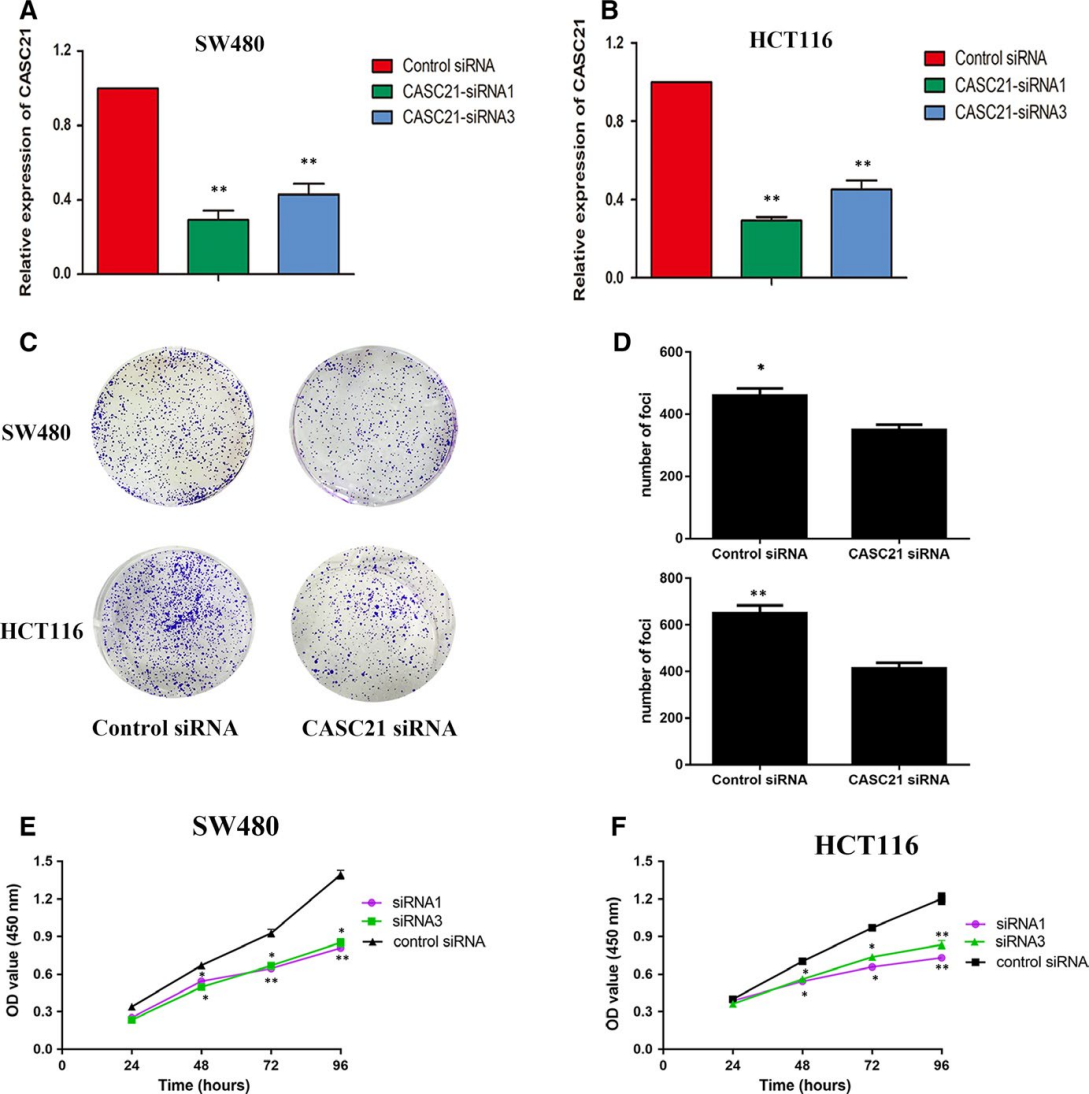
Knockdown of CASC21 impairs colon cancer cell growth in vitro. A, qRT‐PCR assay was used to access the expression of CASC21 after transfecting SW480 with CASC21siRNA, and data were presented as fold change values in cells transfected with CASC21siRNA relative to CASC21 negative controls. B, CASC21 expression was evaluated in HCT116 cells transfected with CASC21siRNA and CASC21 negative controls. C and D, Knockdown of CASC21 significantly impaired the capacity of colony formation in HCT116 and SW480 cells by colony formation assay. E and F, CCK8 assay was performed to test the efficacy of CASC21 silence on cell proliferation in HCT116 and SW480 cells. (*P < .05, **P < .01)

**Figure 4:**
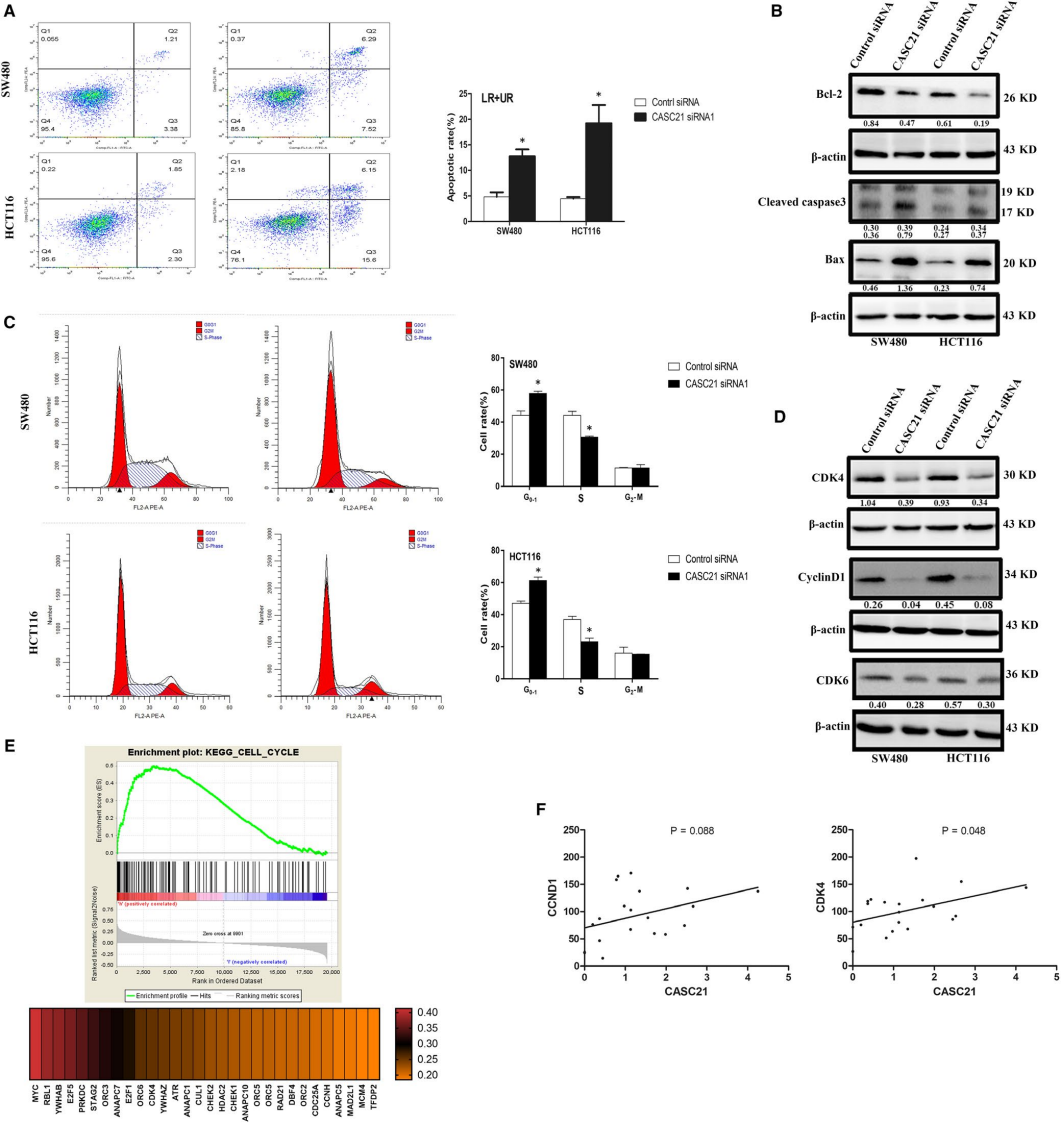
CASC21 knockdown induces cell apoptosis and G1 arrest in colon cancer. A, Flow cytometry was used to detect the apoptotic rates (LR + UR) of cells. LR, early apoptotic cells; UR, terminal apoptotic cells; B, The expression of apoptosis‐relative proteins was analysed by Western blot assays; C, Flow cytometry was used to detect the proportion of cells in S, G1 and G2‐M phases; D, The expression of cell cycle‐related proteins was examined by Western blot assays; E, GSEA analysis showed cell cycle signalling pathway was significantly activated in the CASC21 high expression group; F, The relationship between of CASC21 expression and CDK4 or CCND1 expression was determined by RNA sequencing in 20 colon cancer tissues. Quantitative analysis of photographs from WB was performed by IPP software. (*P < .05, **P < .01)

As expected, CASC21 expression was down‐regulated after CASC21 knockdown by transfecting SW480 and HCT116 cells with CASC21 siRNA (Figure 3A,B). The colony formation assay indicated that CASC21 knockdown inhibited the ability of colony formation in both SW480 and HCT116 cells (Figure 3C,D). CCK8 assay also confirmed that knockdown of CASC21 suppressed cell proliferation in SW480 and HCT116 cells (Figure 3E,F). Cell proliferation assays including CCK8 and colony formation assays emphasized the possibility that CASC21 might be associated with tumour growth in colon cancer. Apoptosis assays showed that SW480 and HCT116 cells transfected with CASC21siRNA had higher apoptotic rates than negative controls (Figure 4A). The protein expression levels of cleaved Caspase‐3 and bax were increased while bcl‐2 was decreased in HCT116 and SW480 cells transfected with CASC21siRNA (Figure 4B). Furthermore, CASC21 knockdown significantly decreased the number of cells in S phase while increasing cells in G0/G1 phase compared with negative controls (Figure 4C). Western blot assays showed that CASC21 knockdown markedly reduced the protein expression levels of CDK4, CDK6 and cyclin D1 (Figure 4D). To further clarify the impact of CASC21 on cell proliferation, we performed the gene set enrichment analysis (GSEA). We found that CASC21 co‐expressed with cell cycle‐related genes and cell cycle pathway was significantly activated in CASC21 high expression group (Figure 4E). Furthermore, we also proved the positive relationships between the expression levels of CASC21 and co‐expressed cell cycle‐related genes including CCND1 and CDK4 by RNA sequencing using 20 colon cancer tissues (Figure 4F). We uploaded the results of RNA‐sequencing analysis in Table S3.

### 3.4 CASC21 promotes cell migration and invasion by activating WNT/β‐catenin signalling in colon cancer

**Figure 5:**
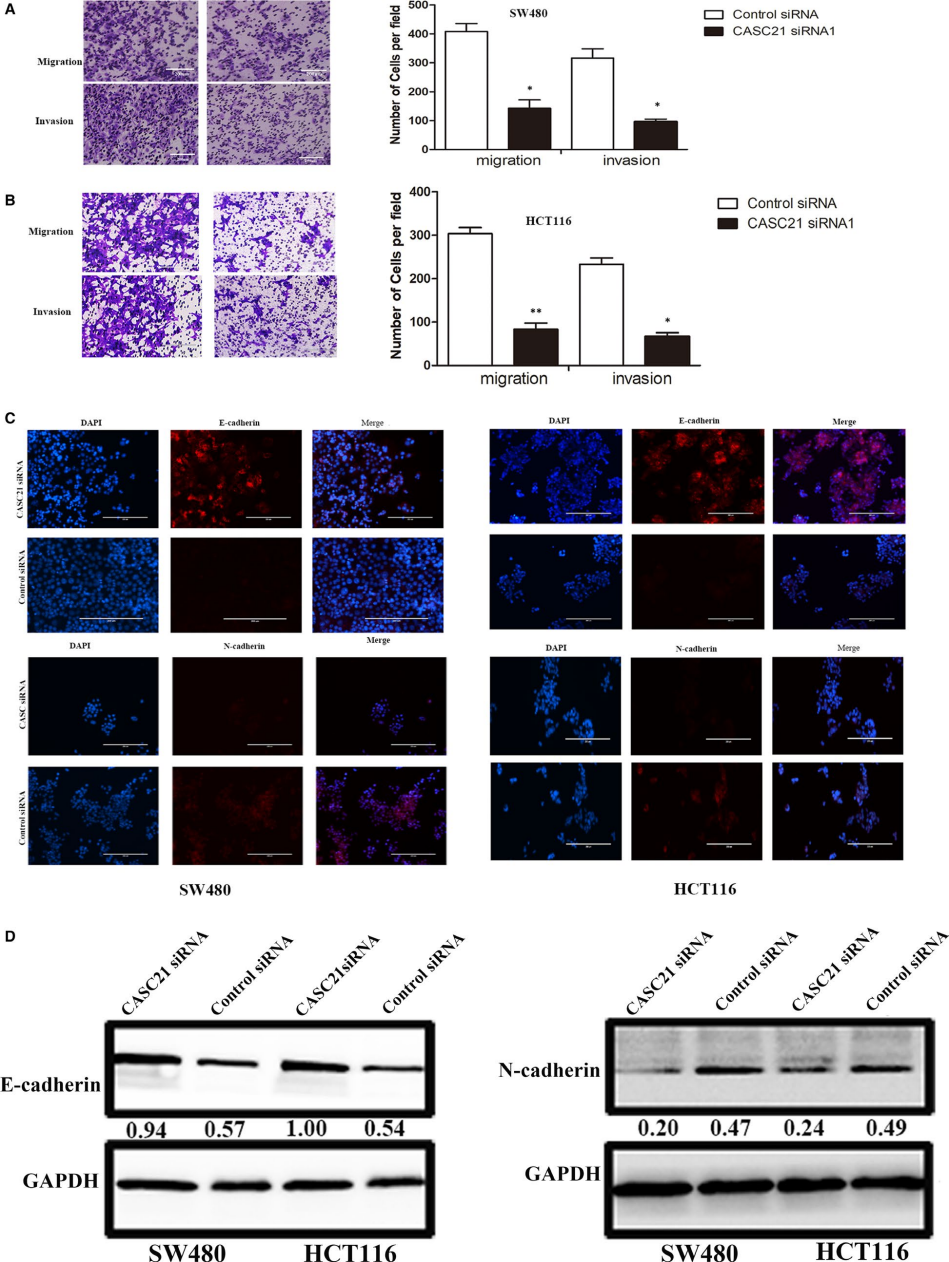
CASC21 knockdown inhibits colon cancer cell invasion and migration in vitro. A and B, Transwell assays demonstrated that CASC21 knockdown inhibited migration and invasion in HCT116 and SW480 cells; C, Immunofluorescence analysis showed knockdown of CASC21 significantly inhibited the expression of N‐cadherin while increasing the expression of E‐cadherin; D, Western blot showed the protein expression of N‐cadherin was decreased and E‐cadherin expression was increased after CASC21 knockdown in HCT116 and SW480 cells. Quantitative analysis of photographs from WB was performed by IPP software. (*P < .05, **P < .01)

**Figure 6:**
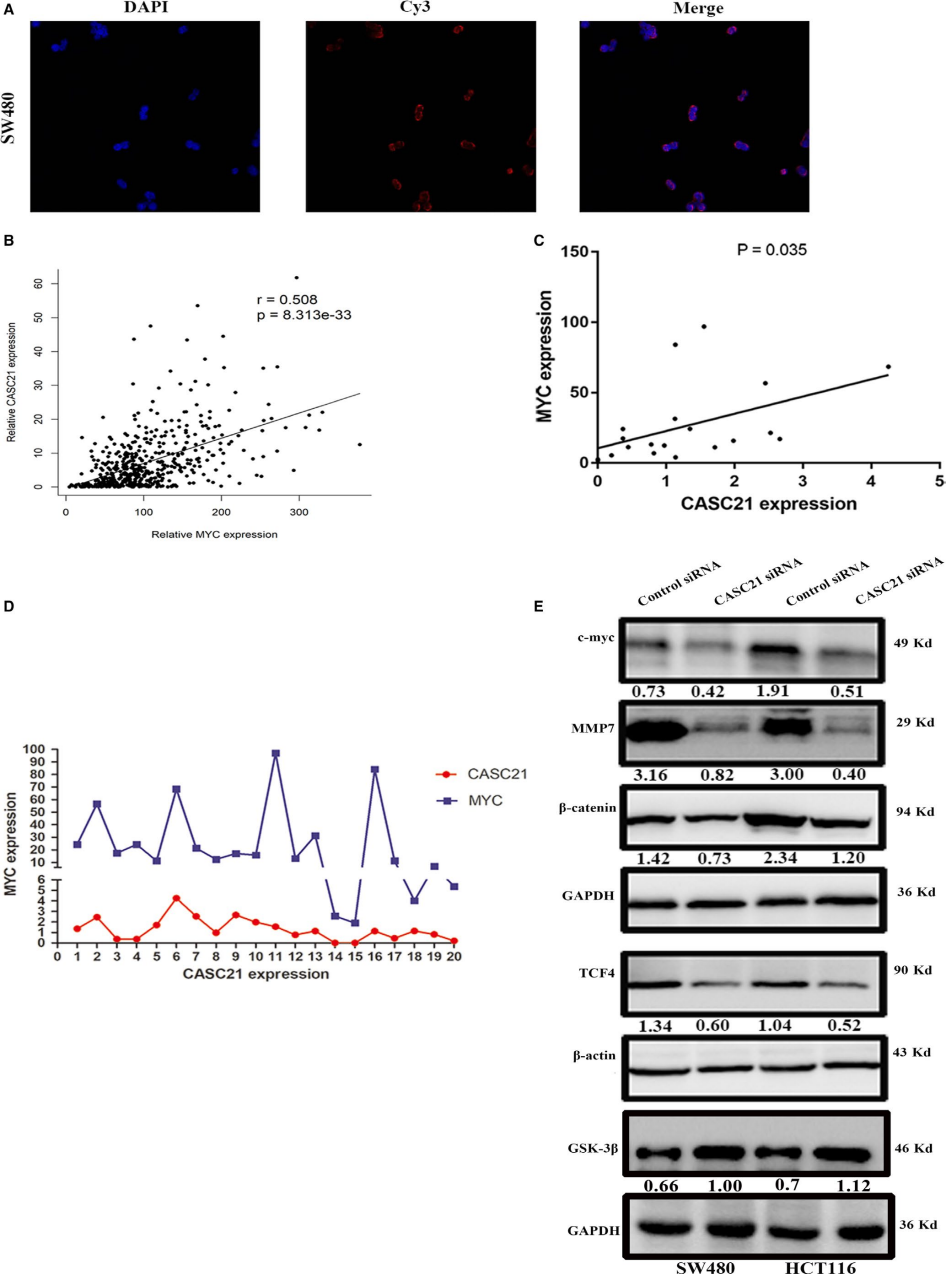
CASC21 promotes tumour metastasis by activating WNT/β‐catenin signalling in colon cancer. A, FISH analysis showed the subcellular location of CASC21 (red) in the cytoplasm and nuclear fractions of SW480 cells; B, TCGA data showed a positive relationship between CASC21 and MYC expression in colon cancer; C, Our RNA‐seq analysis cohort showed the association between CASC21 and MYC expression by Spearman correlation analysis. D, The expression levels of CASC21 and MYC were determined using 20 colon cancer tissues; E, The protein expression of β‐catenin, c‐Myc, TCF‐4, MMP7 and GSK3β in HCT116 and SW480 cells was detected by Western blot. Quantitative analysis of photographs from WB was performed by IPP software

Since CASC21 expression was increased in patients with III‐IV stage, we investigated whether CASC21 affected cell invasion and migration in colon cancer. As a result, CASC21 knockdown significantly inhibited the capacity of migration and invasion in SW480 and HCT116 cells (Figure 5A,B). Immunofluorescence assay showed that CASC21 silence decreased N‐cadherin expression while increasing E‐cadherin expression (Figure 5C). Similar results from Western blot assay were presented in Figure 5D. To investigate the potential mechanism of CASC21, we detected the subcellular location of CASC21. According to FISH assay, CASC21 mainly located in cytoplasm compared with nuclear (Figure 6A). Thus, we hypothesized CASC21 might exert its function by modulating mRNA. It is reported that a lncRNA can regulate the expression levels of genes located around it. CASC21 was located around MYC. In this study, we found a positive relationship between CASC21 and MYC expression in TCGA and our RNA‐sequencing cohorts (Figure 6B,D). At the protein level, CASC21 knockdown decreased the protein expression of c‐Myc (Figure 6E).

According to previous reports, WNT/β‐catenin signalling activation plays an important role in regulating epithelial‐mesenchymal transition (EMT) process and leading to tumour metastasis. Several cancer‐related lncRNAs located in 8q24.21 locus were involved in WNT/β‐catenin signalling abnormal activation; thus, it is possible that CASC21 promotes EMT through activating WNT/β‐catenin signalling. To test our hypothesis, we detected the protein expression levels of WNT pathway targets. And it turns out that CASC21 knockdown decreased the protein expression levels of cyclin D1, TCF‐4, MMP7 and β‐catenin (Figure 6E). Because GSK3β is a crucial regulator of WNT/β‐catenin signalling pathway, and GSK3β is involved in destabilizing β‐catenin, we detected whether CASC21 knockdown affected GSK3β expression, and the results showed that CASC21 knockdown elevated GSK3β protein expression (Figure 6E). Collectively, CASC21 might promote EMT by activating WNT/β‐catenin signalling in colon cancer.

### 3.5 CASC21 knockdown inhibits colon cancer cell tumorigenesis and metastasis in vivo

**Figure 7:**
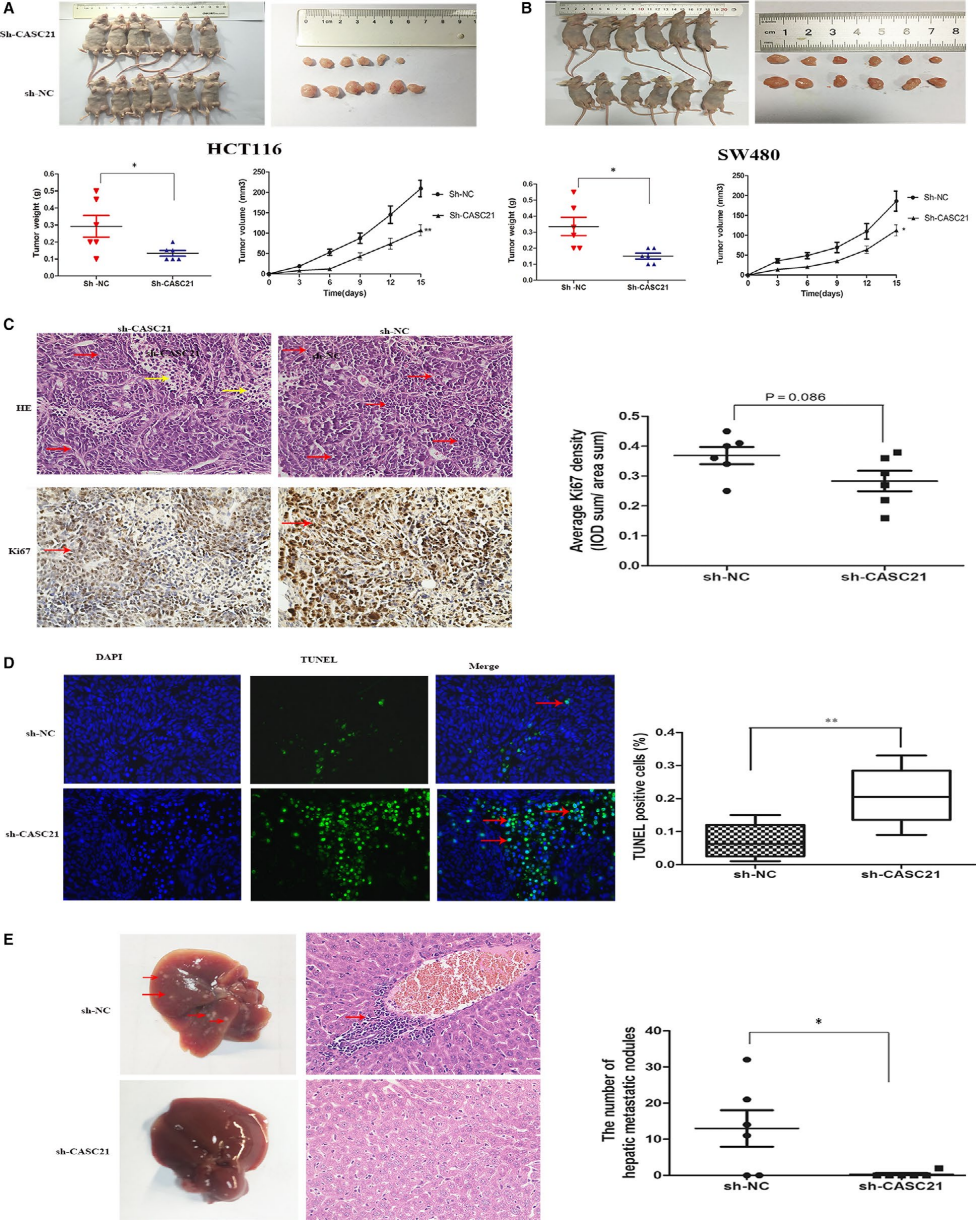
CASC21 knockdown inhibits tumour growth and metastasis in vivo. A, Effect of CASC21 on tumour formation in a nude mouse xenograft model. Representative image of tumours in mice injected with sh‐CSAC21‐HCT116 cells or negative controls; B, Effect of CASC21 on tumour formation in a nude mouse xenograft model. Representative image of hepatic metastasis in mice injected with sh‐CSAC21‐SW480 cells or negative controls C, Ki67 expression in tumour tissues from sh‐CASC21 or negative control group was determined by immunohistochemistry. Quantitative analysis of ki67 expression was performed by IPP software. Haematoxylin and eosin staining showed tumour sites (red arrows) and adjacent normal sites (yellow arrows), respectively. D, TUNEL staining was used to detect apoptotic cells, and red arrows (green dots) represented apoptotic cells. (Scale bars = 100 μm); E, Efficacy of CASC21 on tumour metastasis in a hepatic metastasis mouse model. Representative image of hepatic metastasis in mice injected with sh‐CSAC21‐SW480 cells or negative controls (Scale bars = 100 μm). *P < .05, ** *P* < .01

To elucidate whether CASC21 promotes tumour growth and metastasis in vivo, we established tumorigenicity and metastasis models, respectively. We observed that the average size and weight of tumours were significantly decreased in sh‐CASC21 group than negative control group (Figure 7A,B). IHC assay showed that Ki‐67 expression was decreased in sh‐CASC21 tumours compared with control tumours (Figure 7C), indicating CASC21 knockdown inhibited tumour growth in vivo. Moreover, TUNEL staining test showed tumours in sh‐CASC21 group exhibited more apoptotic cells (Figure 7D). Besides, we assessed the role of CASC21 in affecting tumour metastasis and found the number of hepatic metastatic nodules was decreased in sh‐CASC21 tumours than negative controls (Figure 7E). Taken together, we suggest that CASC21 promotes colon cancer growth and metastasis in vivo.

## 4 DISCUSSION

Recent studies have revealed that lncRNAs are crucial regulators in tumour initiation and metastasis by multiple mechanisms.^16^, ^17^, ^18^ The research of lncRNAs requires a combination of bioinformatics‐, molecular biology‐ and genetics‐based approaches. In the present study, we identified DElncRNAs in colon cancer, and some of them were validated by previous reports. CASC21 was one of the 50 most up‐regulated lncRNAs, and the special position of CASC21 on the chromosome attracted our attention. CASC21 is a novel lncRNA, and its clinical significance has not been revealed, to date. Here, we first proved CASC21 is significantly up‐regulated and observed a positive relationship between CASC21 expression and TNM classification in colon cancer, implying its potential clinical significance in colon cancer. Moreover, the surprise is that CASC21 expression was slightly higher in patients with pMMR status. Ozawa T et al reported a trend towards higher expression of CCAT2 in patients with microsatellite stable (MSS) colorectal cancer (CRC) compared with microsatellite instable‐high (MSI‐H) CRC.^14^ Therefore, our findings supported our previous hypothesis that CASC21 might act as an oncogene in colon cancer.

MYC, contributing to tumorigenesis, is a proto‐oncogene. It is reported that the 8q24 region harbours MYC proto‐oncogene and several MYC‐related lncRNAs including CCAT1, CCAT2 and CASC11. Similarly, CASC21 is located in the 8q24 locus and we observed a positive relationship between CASC21 and MYC expression, providing the evidence for its potential oncogenic role in colon cancer. Our in‐depth research confirmed CASC21 had an obvious tumorigenic impact in colon cancer. In vivo, CASC21 enhanced the capacity of cell proliferation in CCK8 and colony formation assays as well as cell invasion and migration in transwell assay. In vitro, CASC21 promoted tumour growth and metastasis in animal experiments. Moreover, we found that CASC21 knockdown decreased protein expression levels of N‐cadherin while increasing E‐cadherin expression, suggesting CASC21 might affect tumour metastasis by promoting EMT process. WNT/β‐catenin signalling abnormal activated is one of the majority causes leading to various human cancers including colon cancer.^19^, ^20^ WNT signalling also induces EMT process and finally promotes tumour metastasis.^21^ As mentioned previously, CCAT2, located at 8.24.21, was proved to be involved in tumorigenicity through activating WNT/β‐catenin signalling. We observed CASC21 located around CCAT2; thus, we speculated that CSAC21 might have the similar biological function to CCAT2 by activating WNT/β‐catenin signalling. In this study, we found CASC21 knockdown obviously decreased the protein expression levels of WNT/β‐catenin signalling targets. GSK3β plays a crucial role in regulating β‐catenin. Our findings indicated CASC21 knockdown increased the GSK3β expression. These results confirm that CASC21 silence may increase GSK3β expression and then reduce WNT/β‐catenin signalling activation.

Up to now, the detailed mechanisms of dysregulated lncRNAs in cancer have not been interpreted completely. Here, we attempted to predict the molecular mechanisms of DElncRNAs using the ceRNA network and provided possible approaches for experimental verification. In our previous work, we have demonstrated lncRNA KCNQ1OT1 promoted EMT in colorectal cancer (CRC) by sponging miRNA217,^22^ and the KCNQ1OT1‐miRNA217 axis was included in the ceRNA network we built in this study.

In conclusion, our work first proves CASC21 may serve as an oncogene by activating WNT/β‐catenin in colon cancer. Furthermore, we provide a great number of DElncRNAs and a ceRNA network for further study in colon cancer. Although CASC21 promotes colon cancer growth and metastasis by activating WNT/β‐catenin signalling, there are more experiments required to clarify it clearly.

## CONFLICTS OF INTERESTS

No conflict of interests.

## AUTHORS’ CONTRIBUTIONS

Xiaoping Qian and Baorui Liu designed the study. Qun Zhang and Yinzhu Bian prepared manuscript drafting and performed the in vitro experiments. Yiping Zhu and Li Wan contributed to the in vivo experiments. All authors reviewed and revised this manuscript.

## Data Availability

The data that support the findings of this study are openly available in the supplementary materials section.
