# Supplementary figures and images for: Integrative analysis and validation of dysregulated long non‐coding RNAs in colon cancer
Source: J Cell Mol Med. 2020 Jan 20;24(4):2610–21. doi: 10.1111/jcmm.14974 (PMC7028851; doi:10.1111/jcmm.14974)

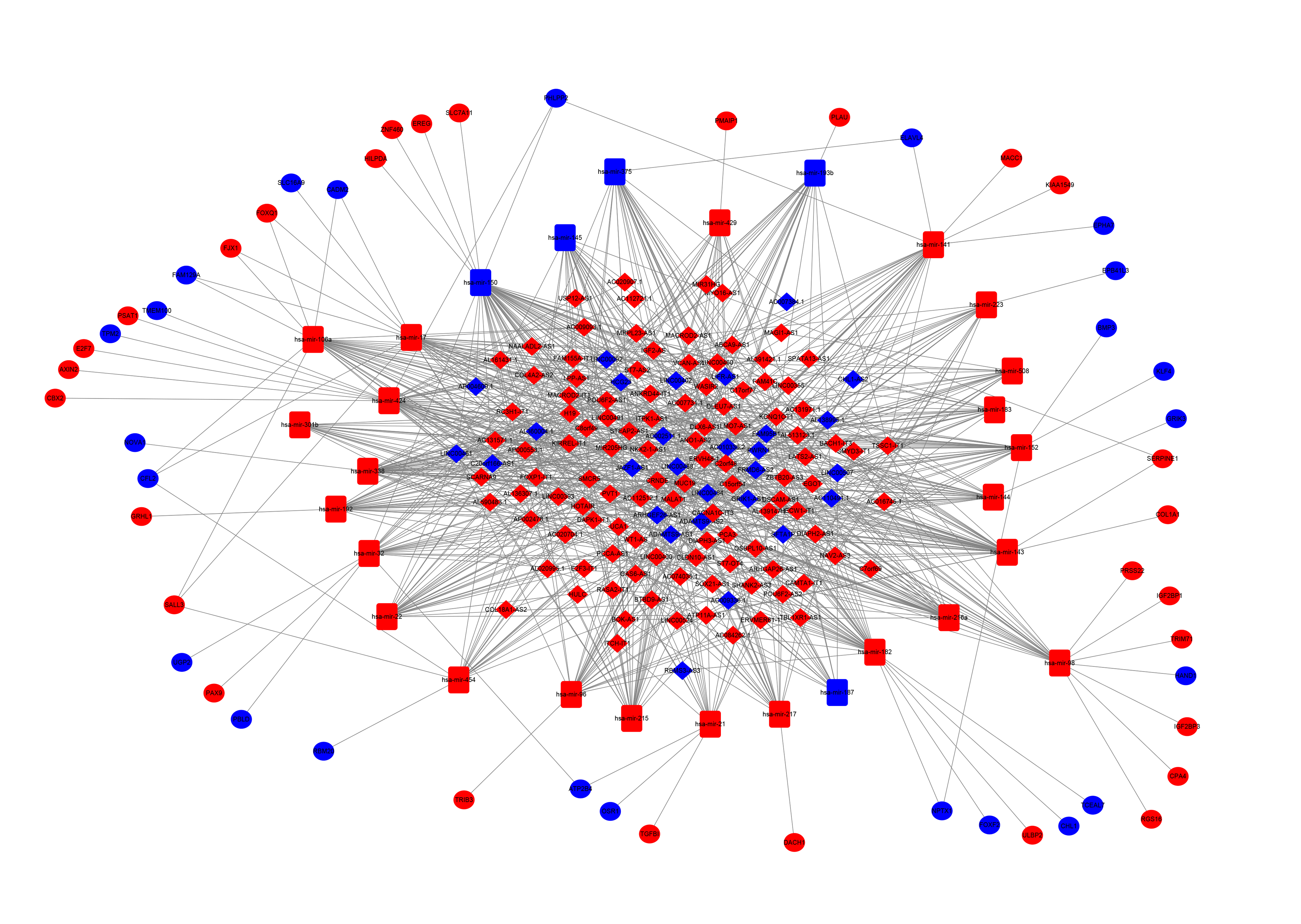

Supplement: Supplementary file 1 [file JCMM-24-2610-s001.tif]

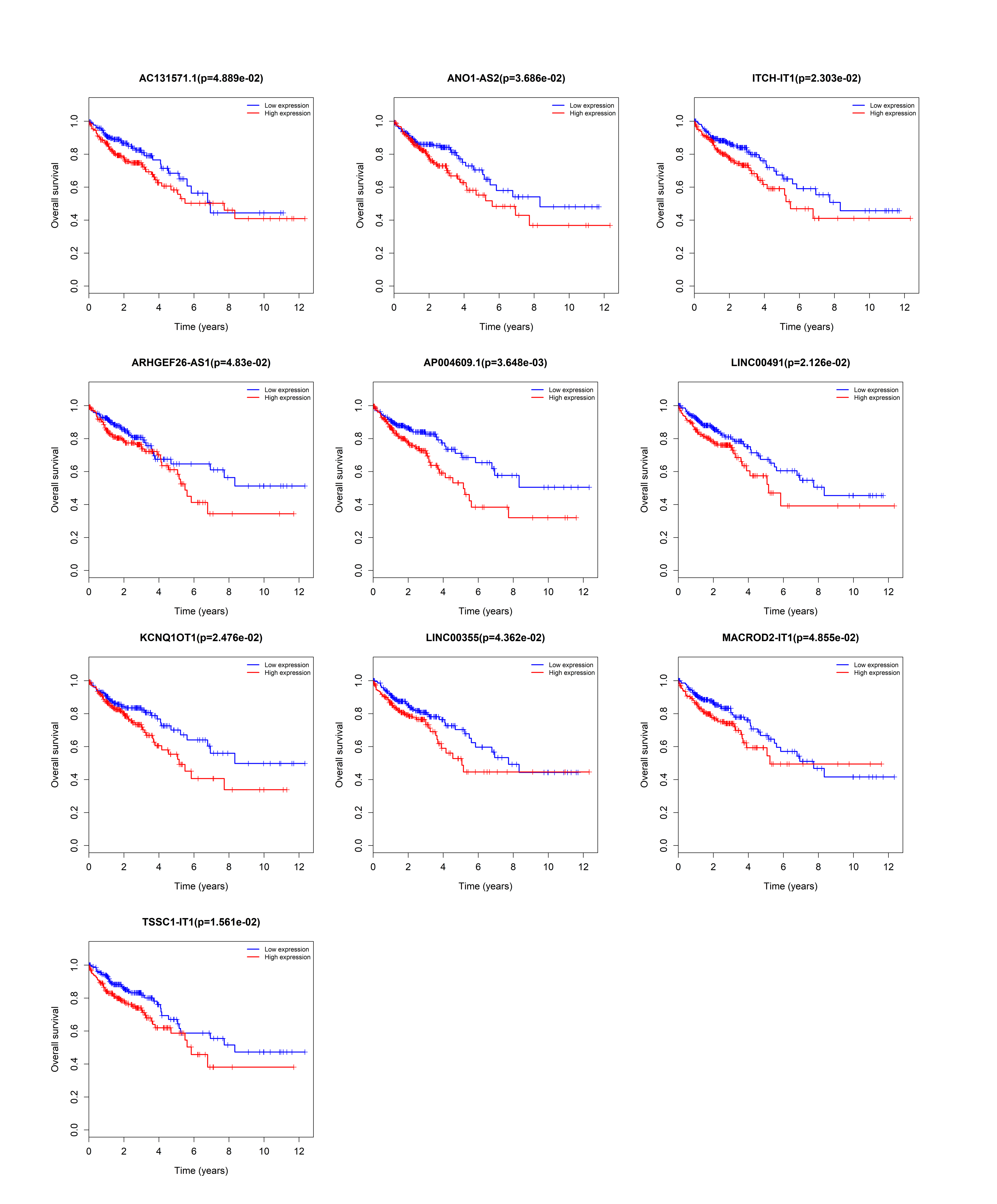

Supplement: Supplementary file 2 [file JCMM-24-2610-s002.tif]

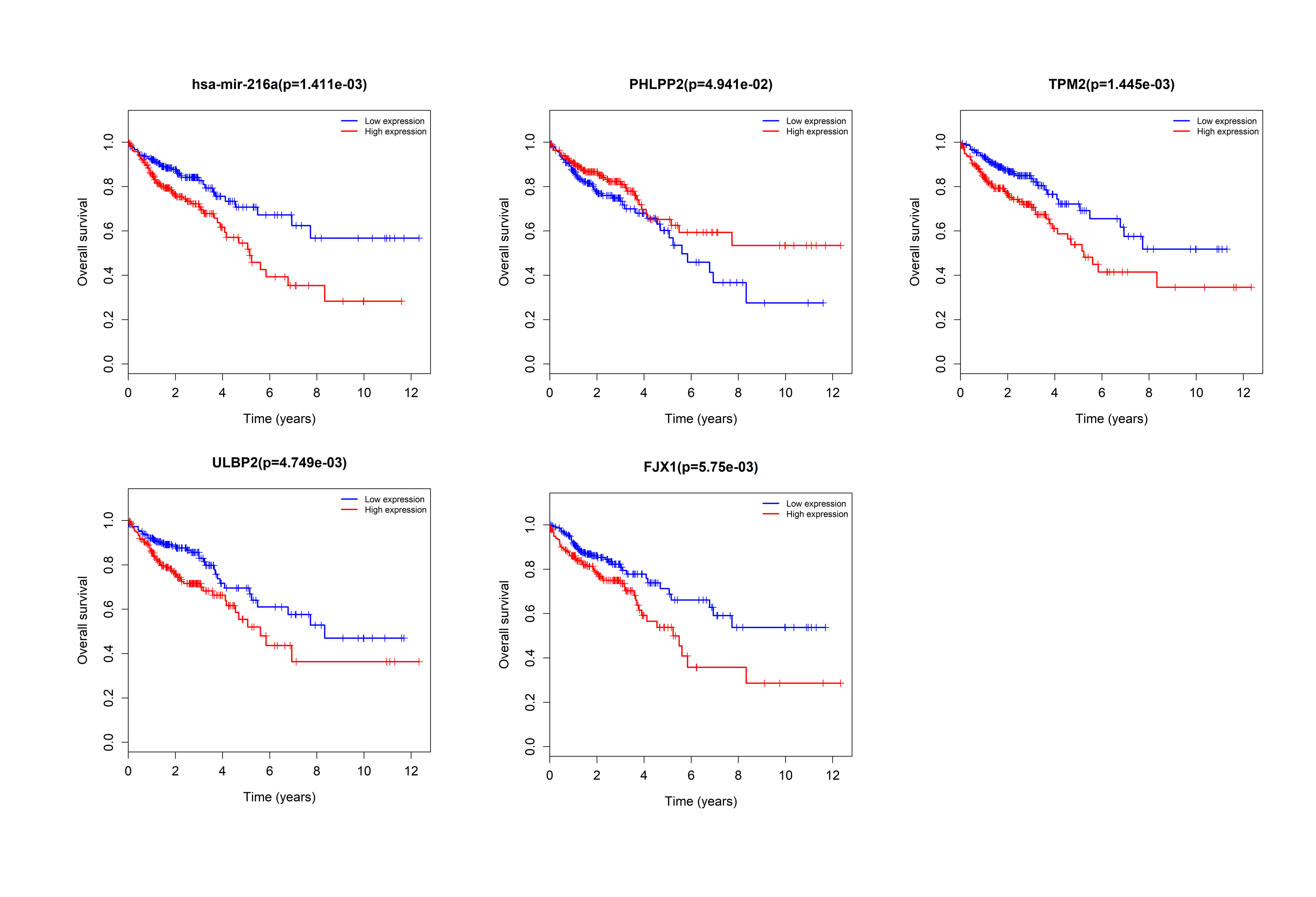

Supplement: Supplementary file 3 [file JCMM-24-2610-s003.tif]
