# Supplementary material for: Integrative analysis and validation of dysregulated long non‐coding RNAs in colon cancer
Source: J Cell Mol Med. 2020 Jan 20;24(4):2610–21. doi: 10.1111/jcmm.14974 (PMC7028851; doi:10.1111/jcmm.14974)
Supplement: Supplementary file 5 [file JCMM-24-2610-s005.docx]

**Table S3:** A list of top 20 enriched KEGG pathways of DEmRNAs in the ceRNA network.

| Term | ID | P-Value |
| --- | --- | --- |
| Signaling pathways regulating pluripotency of stem cells | hsa04550 | 0.000525 |
| MicroRNAs in cancer | hsa05206 | 0.004265 |
| Vitamin B6 metabolism | hsa00750 | 0.007538 |
| Adrenergic signaling in cardiomyocytes | hsa04261 | 0.01166 |
| Axon guidance | hsa04360 | 0.015924 |
| Galactose metabolism | hsa00052 | 0.034011 |
| Pentose and glucuronate interconversions | hsa00040 | 0.039222 |
| Glycine, serine and threonine metabolism | hsa00260 | 0.043371 |
| Amino sugar and nucleotide sugar metabolism | hsa00520 | 0.051616 |
| PI3K-Akt signaling pathway | hsa04151 | 0.05332 |
| Endometrial cancer | hsa05213 | 0.055713 |
| Basal cell carcinoma | hsa05217 | 0.058775 |
| Starch and sucrose metabolism | hsa00500 | 0.06081 |
| Colorectal cancer | hsa05210 | 0.06588 |
| Pertussis | hsa05133 | 0.078936 |
| Biosynthesis of amino acids | hsa01230 | 0.078936 |
| Cardiac muscle contraction | hsa04260 | 0.081924 |
| Complement and coagulation cascades | hsa04610 | 0.082918 |
| ECM-receptor interaction | hsa04512 | 0.085893 |

KEGG Kyoto Encyclopedia of Genes and Genomes, DEmRNA differentially expressed mRNA, ceRNA competing endogenous RNA
